# Fiducial markers visibility and artefacts in prostate cancer radiotherapy multi-modality imaging

**DOI:** 10.1186/s13014-019-1447-1

**Published:** 2019-12-26

**Authors:** Sarah O. S. Osman, Emily Russell, Raymond B. King, Karen Crowther, Suneil Jain, Cormac McGrath, Alan R. Hounsell, Kevin M. Prise, Conor K. McGarry

**Affiliations:** 1 Centre of Cancer Research and Cell Biology, Queen’s University Belfast, Belfast, Northern Ireland BT7 1NN UK; 2 Radiotherapy Physics, Northern Ireland Cancer Centre, Belfast Health and Social Care Trust, Belfast, UK; 3 Radiotherapy Department, Northern Ireland Cancer Centre, Belfast Health and Social Care Trust, Belfast, UK; 4 Clinical Oncology, Northern Ireland Cancer Centre, Belfast Health and Social Care Trust, Belfast, UK; 5 Radiological Sciences and Imaging, Belfast Health and Social Care Trust, Forster Green Hospital, Belfast, UK

**Keywords:** IGRT, Prostate Cancer, Pelvic phantom, Fiducial markers, Artefacts, Multi-modality imaging

## Abstract

**Background:**

In this study, a novel pelvic phantom was developed and used to assess the visibility and presence of artefacts from different types of commercial fiducial markers (FMs) on multi-modality imaging relevant to prostate cancer.

**Methods and materials:**

The phantom was designed with 3D printed hollow cubes in the centre. These cubes were filled with gel to mimic the prostate gland and two parallel PVC rods were used to mimic bones in the pelvic region. Each cube was filled with gelatine and three unique FMs were positioned with a clinically-relevant spatial distribution. The FMs investigated were; Gold Marker (GM) CIVCO, GM RiverPoint, GM Gold Anchor (GA) line and ball shape, and polymer marker (PM) from CIVCO. The phantom was scanned using several imaging modalities typically used to image prostate cancer patients; MRI, CT, CBCT, planar kV-pair, ExacTrac, 6MV, 2.5MV and integrated EPID imaging. The visibility of the markers and any observed artefacts in the phantom were compared to in-vivo scans of prostate cancer patients with FMs.

**Results:**

All GMs were visible in volumetric scans, however, they also had the most visible artefacts on CT and CBCT scans, with the magnitude of artefacts increasing with FM size. PM FMs had the least visible artefacts in volumetric scans but they were not visible on portal images and had poor visibility on lateral kV images. The smallest diameter GMs (GA) were the most difficult GMs to identify on lateral kV images.

**Conclusion:**

The choice between different FMs is also dependent on the adopted IGRT strategy. PM was found to be superior to investigated gold markers in the most commonly used modalities in the management of prostate cancer; CT, CBCT and MRI imaging.

## Introduction

A large proportion of patients diagnosed with prostate cancer are treated with external beam radiation therapy (EBRT). Currently, the standard of care of EBRT for prostate cancer in the United Kingdom is to deliver 60 Gy in 20 fractions, using intensity modulated radiation therapy (IMRT). Due to the low alpha/beta ratio for prostate cancer [1], i.e. the sensitivity of prostate cancer to the dose per fraction rather than the total dose, there is more interest now in prostate hypo-fractionation with many clinical trials delivering 40–50 Gy in 5 fractions [2, 3]. There is also an increased interest in boosting dominant intra-prostatic lesions (DIL) to higher doses [4]. Delivering high doses is made possible by the introduction of IMRT, rotational techniques such as volumetric modulated arc therapy VMAT (VMAT), and with the adoption of image guided radiation therapy (IGRT) strategies.

A major concern in highly focussed, intensity modulated hypo-fractionated treatments is the uncertainty associated with reproducibility of daily patient setup (inter-fraction setup) and during the treatment (intra-fraction setup). Setup accuracy is particularly crucial in both moderate and extreme hypo-fractionation settings. Failure to deliver the high doses, with their associated sharp dose gradients, can have severe implications in local control (missing the tumours) or in introducing toxicity when delivering the high dose to neighbouring organs at risk. Intra-fraction uncertainties (e.g. due to internal organ movements - rectum and bladder filling changes - or patient movement) may be reduced by shortening treatment times [5]. Inter-fraction setup errors are usually reduced and controlled using IGRT.

Several IGRT strategies can be adopted to increase accuracy in the daily setup of the patient [6]. Typical IGRT workflows for conventional linear accelerators (linacs; X-ray or CBCT-guided) are shown in Additional file 1: Figure S1. Workflow of MRI-guided radiotherapy IGRT on MRI linacs was recently presented by Kerkmeijer et al. [7]). The accuracy of IGRT strategies is determined by the imaging modality employed and the matching technique adopted (e.g. bone or soft tissue match). Despite the theoretical superiority of soft-tissue matching for prostate, the practicality of matching the prostate daily with the patient on the treatment couch might be challenging due to the suboptimal contrast of soft tissue. Therefore, matching to soft tissue can increase treatment time and subsequently increase the risk of intra-fraction errors [5]. Moreover, matching to soft-tissue is also more prone to larger inter- and intra-observer variability [8].

Surgically inserted intra-prostatic fiducial markers (FMs) have been widely used for prostate cancer IGRT to improve contrast and to provide a fast and accurate method to setup patients. A recent review on the subject can be found in reference [9]. FMs are well tolerated, safe and effective as reported by a number of relatively large studies [10–12]. FMs are also useful for the registration (matching) of MRI and CT for organ delineation. This is especially relevant when outlining dominant intra-prostatic lesions (DIL) [4] and, as observed in our institution, in delineating anatomy modifiers (e.g. SpaceOAR) [13]. FM-based registration allows the fast and accurate assessment of anatomical changes that might require interventions on a daily (or weekly) basis using kV and/or MV imaging [14]. Furthermore, FM-based IGRT has also allowed the reduction of CTV-PTV margins [15, 16]. Traditionally, three intra-prostatic non-co-linear FMs are used for each patient to provide a triangulation required for the measurement of position in different planes i.e. accurate 3D set-up of patients [17].

An ideal fiducial marker should be visible in all imaging modalities of interest with the least alteration to image quality (artefacts) possible. Minimizing the effects of artefacts on planning CT scans is especially important as artefacts may interfere with structure delineation and dose calculation accuracy [18, 19]. There have been several reports from experimental results of dose perturbations from gold fiducial markers [19]. Chow et al. reported a 21% increase in dose from 6 MV photon beams and up to 22% decrease in dose distal to seeds [19]. These effects are even more severe in proton treatments [20]. Traditionally, gold fiducial markers have been used and there are different shapes and sizes available from different manufacturers. However, commercially available markers of different materials, designs and sizes now exist in the market. The goal of this study was to investigate the visibility as well as the associated artefacts of different FMs used for IGRT for prostate cancer patient treated with EBRT. This current work builds on previous published studies while addressing some of the highlighted limitations, e.g. the use of Superflab to embed the FMs with inevitable air gaps, phantoms that contain materials that are not MRI compatible and gel phantoms that can only represent soft tissue characteristics [21–23].

In this study, a novel pelvic phantom that mimics a patient’s pelvis (soft-tissue and bone) was developed and tested. A direct comparison was made between the visibility of FMs and the presence artefacts for three different types of FMs in-vivo (on patients’ scans) and on the in-house built pelvic phantom when using multiple imaging modalities.

Moreover, a comparison was made between four different types of commercially available FMs for prostate cancer radiotherapy in the phantom study. The most commonly used IGRT imaging modalities and the use of different acquisition protocols were investigated. To test inter-scanner variabilities, the phantom was also scanned on multiple CT and MRI scanners using similar scanning protocols.

## Materials and methods

### Phantom design

**Fig. 1:**
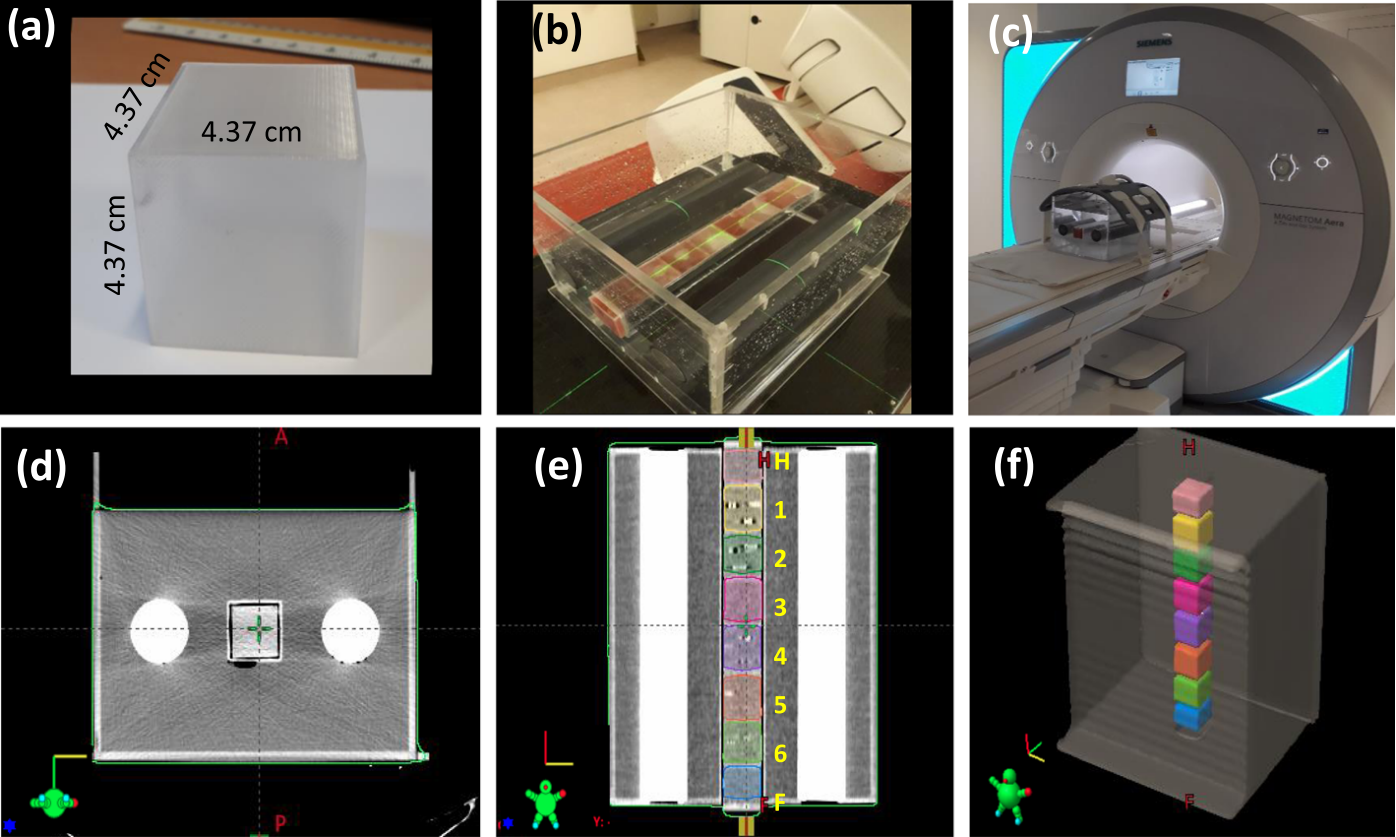
**a** 3D printed boxes used to house the gel with or without FMs (**b**) the complete pelvic phantom being filled with water and setup for scanning on a Varian TrueBeam (**c**) Pelvic phantom setup on a Siemens Aera 1.5 T MRI scanner. Panels d--f show CT scans of the pelvic phantom (**d**) transverse view, (**e**) coronal view showing boxes 1–6 and Head (H) and feet (F) boxes with no FMs, and (**f**) 3D view of the structures outlined on Varian Eclipse treatment planning system)

To facilitate this study, a novel cubical PMMA pelvic water phantom 33.2 × 30 × 26 cm^3^ (*length×width×height*) was constructed, see Fig. 1 . Modifying the design of a previously reported test-phantom by Radford et al. [24], the phantom contained two 50 mm diameter rods of high-density polyvinyl chloride (PVC) material to mimic bone and had a parallel square hollow tube at the centre. Eight 4.4 × 4.4 × 4.4 cm^3^ hollow cubes were 3D printed from polyethylene terephthalate glycol-modified (PETG) filament using an X400 German RepRap fused filament fabrication (FFF) dual-extrusion 3D printer (German RepRap GmbH, Feldkirchen, Germany). Each cube was filled with gel (edible gelatine - inspired by phantoms using agar-based gels [22, 25]) and 3 fiducial markers were placed with a spatial distribution similar to prostate implanted FMs. To determine the typical FMs spatial distribution within the prostate used at our centre, CTs for six patients with implanted fiducials were retrospectively analysed. For each patient the x, y and z coordinates were determined for the three implanted fiducials. The coordinates for the individual fiducials were corrected to the average position of all three fiducials to allow the calculation of an average marker distribution for the six patients.

An initial study was conducted to assess the potential migration of FMs over time. One box containing 3 FMs implanted in gel was repeatedly scanned. The position of the individual FMs relative to the box and to each other was determined and tracked over a 4 week period.

In this study, eight 3D printed boxes were filled with gel, with or without FMs. Before filling the boxes containing FMs, external markings were used to clearly describe the desired location of the FMs within each box. The boxes were then filled with gel to the lowest specified mark and left to set. Once the gel had set, a marker was placed on the specified position and the box was filled up to the next desired level. After implanting all three fiducials, the boxes were then filled to the top with gel. Four cubes contained unique FMs, one cube had a repeat of one of the markers in a different geometrical configuration, while the sixth cube had a repeat of one of the FMs to check consistency. Two cubes were filled with gel but did not contain any FMs. Before each scan, the 3D printed boxes were arranged in the same orientation and inserted in the hollow tube of the PMMA phantom before the phantom was filled with water. Each 3D printed box therefore represented a prostate with or without FMs as shown in Fig. 1.

### Fiducial markers

Four different types of fiducial markers were assessed in this study; Gold Markers (GM) from CIVCO (diameter×length) 1.2 × 3.0 mm (CIVCO radiotherapy, Coralville, Iowa, USA), GM RiverPoint (RP) 1.2 × 3.0 mm (Riverpoint medical, Portland, OR, USA), line shape Gold Anchor™ (GA) 0.4 × 10.0 mm (Naslund Medical AB, Huddinge, Sweden), and polymer-based marker PolyMark™ (PM) 1.0 × 3.0 mm (CIVCO radiotherapy, Coralville, Iowa, USA). GMs CIVCO, RP and polymer FMs (PM) had relatively similar sizes and shapes while GMs GA had much smaller diameters. GAs can fold into a ball shape when implanted in soft tissue. In this study, the GA were placed in the phantom in their original line shape in one box and in a ball shape in a separate box.

### Imaging

#### Phantom data

As shown in Fig. 1, the boxes containing the gel were numbered 1 through 8 and, for each scan, the order of the boxes within the phantom was changed to ensure the two boxes of interest were always placed at the centre of the phantom. For example, Fig. 1e shows the first setup, where boxes 3 and 4 are the two boxes of interest analysed in the image acquisitions.

The phantom was scanned using the following imaging modalities and acquisition parameters;

##### Planning CT scans

Using, tube voltage of 120 kV and tube current 200 mA and 400 mA on two different CT scanners (Optima CT580 and Discovery CT590 RT), both from GE Medical Systems (Chicago, Illinois, United States). Scans of four different setup arrangements, produced by shifting the boxes inside the phantom, were acquired (16 CT scans in total). Different CT slice thicknesses were acquired (0.625 mm, 1.25 mm, 2.5 mm).

##### Cone beam CT

Scans (CBCT, version 2.5.16) on a Varian TrueBeam® (Varian Medical Systems, Palo Alto, CA) Linac using three pelvis scanning protocols; Varian default pelvis (125 kV, 1080 mAs), the local institution’s optimised pelvis protocol (125 kV, 855 mAs), and also default pelvis obese (140 kV, 1687.5 mAs) for the 4 setup arrangements (12 scans).

##### Kilo-voltage orthogonal beams

(kV-kV pairs) for all setup arrangements (4 pairs of planar images); Pelvis (AP) Anterior-Posterior (85 kV, 15 mAs) and pelvis (LAT) lateral (110 kV, 15 mAs).

##### Integrated EPID imaging

Using Varian TrueBeam® IDU EPID with an array of 1024 × 768 pixels and pixel size of 0.392 mm. This was acquired while delivering 10 × 10 cm^2^, 6 MV, 20 MU segments at static gantry angles of 0^∘^ and at 90^∘^ (4 pairs of images).

##### Mega-voltage orthogonal beams

Using a pair of 2.5MV and 6MV planar images with the Varian TrueBeam® DMI EPID with an array of 1280 × 1280 pixels and pixel size of 0.336 mm at gantry angles 0^∘^ and at 90^∘^.

##### Stereoscopic *X-*ray imaging

Using Brainlab ExacTrac (version 6.2.0) (Munich, Germany) for each setup (4 pairs of planar images); acquired using optimised exposure parameters of 100 kV, 400 mA tube current and 100 ms exposure time.

##### MRI

2D T1-weighted (T1w), Turbo Spin Echo (TSE), 2D T2-weighted (T2w) TSE, and 3D gradient echo (Volumetric Interpolated Breath-hold Examination-VIBE) sequences on a Siemens Magnetom Aera 1.5-T MRI scanner (Siemens Healthcare GmbH, Erlangen, Germany) (16 scans).

##### MRI

T1w TSE, T2w TSE and half Fourier Single Shot TSE breath hold sequences on GE optima 450w 1.5-T MRI scanner (Chicago, Illinois, United States) (12 scans).

Further details of the different scanners and scanning parameters used in this study are presented in Table 1.

**Table 1:** Scanners and scanning parameters used to acquire volumetric images of pelvic phantom, all CBCT were acquires using dynamic gain fluoro kV mode

CT	Manufacturer	Model	Scan Type	Slice thickness (mm)	Pixel spacing (mm)	Focal spot (mm)	kVp	Tube current (mA)	Exposure (mAs)	FoV (mm×mm)		
1	GE	Optima CT580	Helical	0.63, 1.25, 2.5	0.98	0.7	120	200	26	500 × 500		
2	GE	Optima CT580	Helical	2.5	0.98	1.2	120	400	106	500 × 500		
3	GE	Discovery CT590 RT	Helical	2.5	0.98	0.7	120	200	53	500 × 500		
4	GE	Discovery CT590 RT	Helical	2.5	0.98	1.2	120	400	106	500 × 500		
CBCT	Manufacturer	Model	Scan Type (Pelvis)	Slice thickness (mm)	Pixel spacing (mm)	Focal spot (mm)	kVp	Tube current (mA)	Exposure (mAs)	FoV (mm×mm)	kV Filter	Bowtie
5	Varian	TrueBeam	Varian	2	0.91	1	125	80	1074	464.9 × 464.9	Titanium	Half fan
6	Varian	TrueBeam	Medium	2	0.91	1	125	63	845	464.9 × 464.9	Titanium	Half fan
7	Varian	TrueBeam	Obese	2	0.91	1	140	99	1683	464.9 × 464.9	Titanium	Half fan
MRI	Manufacturer	Model	Sequence	Slice thickness (mm)	Slice gap (mm)	TR/TE (ms)	ETL	Flip angle (^o^)	Acquisition matrix	FoV (mm×mm)	Receiver bandwidth (Hz/px)	Number of averages
8	SIEMENS	MAGNETOM Aera 1.5 T	2D T2 TSE	3.5	0.35	4800/94	23	90/160	288 × 384	220 × 220	200	3
9	SIEMENS	MAGNETOM Aera 1.5 T	2D T1 TSE	6	1.2	542/24	3	90/140	336 × 448	359 × 359	185	2
10	SIEMENS	MAGNETOM Aera 1.5 T	3D GR	2	–	7.46/4.77	–	10	307 × 384	420 × 420	325	2
11	GE	Optima MR450w 1.5 T	2D TSE	6	1	556/13.1	4	90/160	224 × 512	360 × 360	122	0.5
12	GE	Optima MR450w 1.5 T	2D SSTSE	2	1	567/98.7	–	90/180	224 × 288	200 × 200	122	0.55
13	GE	Optima MR450w 1.5 T	2D TSE	3.5	0.5	6748/106.7	24	90/160	224 × 384	250 × 250	122	2

### Qualitative assessment

All scans were visually inspected by two independent observers to assess the visibility of the FMs on each image and to qualitatively evaluate the artefacts. To further assess the visibility and artefacts of each FM using different imaging modalities, a line profile was produced for each fiducial markers. These line profiles were directly compared to line profiles generated from patients’ scans.

### Quantitative evaluation of FMs visibility and associated artefacts

For volumetric X-ray scans (CT and CBCT), a region of interest (ROI) for each marker was defined as the box containing 3 FM with 3 mm isotropic inner margin (to avoid any edge effect). Air bubbles were also removed from this ROI. Using high resolution segments, the FMs in each box were contoured using the Eclipse™ treatment planning system (version 13.6) using the automatic thresholding tool. The maximum HU threshold defining the FM was set as the highest HU value inside box ROI. The minimum value for the threshold was determined in Eclipse by manually adjusting the window level to view the FM only. This way an approximate low threshold value was obtained for each marker. Contouring was performed on CT scans with 0.625 mm slice thickness and then transferred to other datasets with different slice thicknesses (1.25 and 2.50 mm scans).

To quantitatively evaluate the visibility of the FM, the contrast-to-noise ratio (CNR) was also calculated [26–28]. For each FM, the mean HU (HU_FM_) and standard deviation (σ_FM_) were compared to background (HU_Gel_) and standard deviation (σ_Gel_). The background HU values (mean value and standard deviation) were determined from the two boxes with gel only (no FM). CNR for each FM was then calculated using the equation:

1 $$ CNR=\frac{\left|{HU}_{FM}-{HU}_{Gel}\right|}{\sqrt{{\sigma_{FM}}^2+{\sigma_{Gel}}^2}} $$

The higher the CNR the more visible the object [26, 27].

To quantify the severity of streak artefact, firstly an artefact volume was defined for each FM by subtracting the FM structure from the box ROI and this artefact volume was then used in the analysis. Similar to the analysis by Huang et al. [20], streak artefact index (SI) was calculated for each ROI. The SI is defined as;

2 $$ SI=\frac{{\left|{HU}_{Max}-{HU}_{Min}\right|}_{Artefact}}{\sigma_{Gel}} $$

where *HU*_*Max*_ and *HU*_*Min*_ are defined as the maximum and minimum HU inside the ROI and *σ*_*Gel*_ is the standard deviation of the HU of the background (gel).

Furthermore, the amount of streak artefact was quantified in 2D on axial slices at the centre of each markers. Using an in-house developed MATLAB script (Version 9.5, 2018b), two artefact 2D ROI were defined; High HU ROI representing areas outside the FM and within box RO with High HU (lower; *HU*_*Gel*_ + 3*σ*_*Gel*_, upper: maximum HU value of specified ROI) and similarly a Low HU ROI representing areas of signal void due to artefact (upper: *HU*_*Gel*_ −  − 3*σ*_*Gel*_, lower: minimum HU value in ROI).

In addition, to quantify the artefacts on MRI scans, FM volumes on MRI were measured and compared to volumes calculated using physical dimensions of the markers.

### Patient data

To assess the typical visibility and associated artefacts of different FMs in-vivo, CT, CBCT and T2w-MRI scans of three representative patients from the SPORT High-Risk Trial (Stereotactic PrOstate RadioTherapy in high-risk localized prostate cancer with or without elective nodal irradiation) were assessed [13] (https://www.hra.nhs.uk/planning-and-improving-research/application-summaries/research-summaries/sport-high-risk-trial/). All (but two) trial patients had CIVCO FM implanted for IGRT. Two of the SPORT trial patients had different FMs (PM, GM GA in a ball shape). A comparison was made with phantom images acquired during this study to confirm that the image quality and artefact structure of the phantom images were an accurate representation of what is observed clinically.

## Results

### Gel and FM stability over time

Monitoring the position of individual FMs over a 4 week period on repeat CT scans revealed only sub-millimetre movements of the FM relative to the cube exterior and each other. This indicated that the use of edible gelatine in our study of FM was reliable for preserving the setup of fiducial markers (the cubes were properly stored between scans 2–4^∘^C). Detailed results from this study can be found in Additional file 1: Figure S2 and Additional file 1: Table ST1).

### Phantom characteristics

**Fig. 2:**
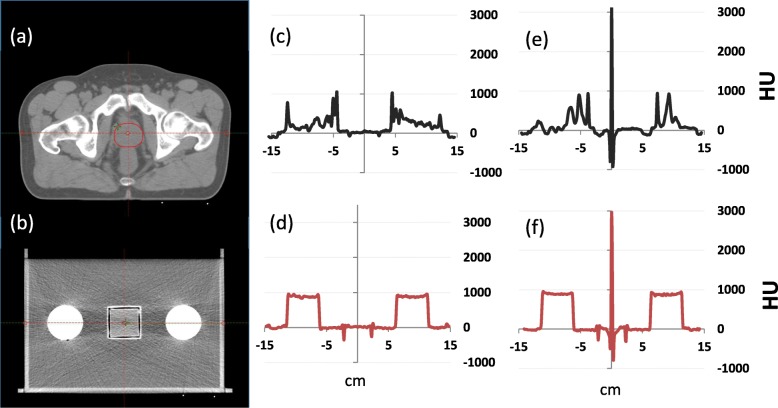
Transverse CT slice of a representative (**a**) prostate cancer patient’s pelvis (**b**) phantom with no FM, (**c**) and (**d**) corresponding lateral line profiles for (**a**) and (**b**) respectively. **e** A line profile from a patient with CIVCO GM. **f** Phantom line profile with CIVCO FM

To characterize the pelvic phantom used in this study, a line profile acquired across an axial CT slice of the phantom and another acquired across an axial CT slice of a prostate cancer patient at the level of the prostate are shown in Fig. 2. The y-scale of the profiles denotes the voxel value for individual voxels across the line profile in Hounsfield units (HU). Maximum HU values for the patient’s bony anatomy and the PVC rods were found to be relatively comparable (1120 HU and 961 HU for the patient and phantom, respectively). HU values for the patients’ soft tissue and the phantoms gel regions were also found to be in good agreement (e.g. average HU of 42 [range:11--73] and 35 [range: 8--90] for the patients’ prostate and the gel contained within the phantom cube, respectively). All line profiles presented in this study were generated in Eclipse TPS.

### Qualitative assessment

**Fig. 3:**
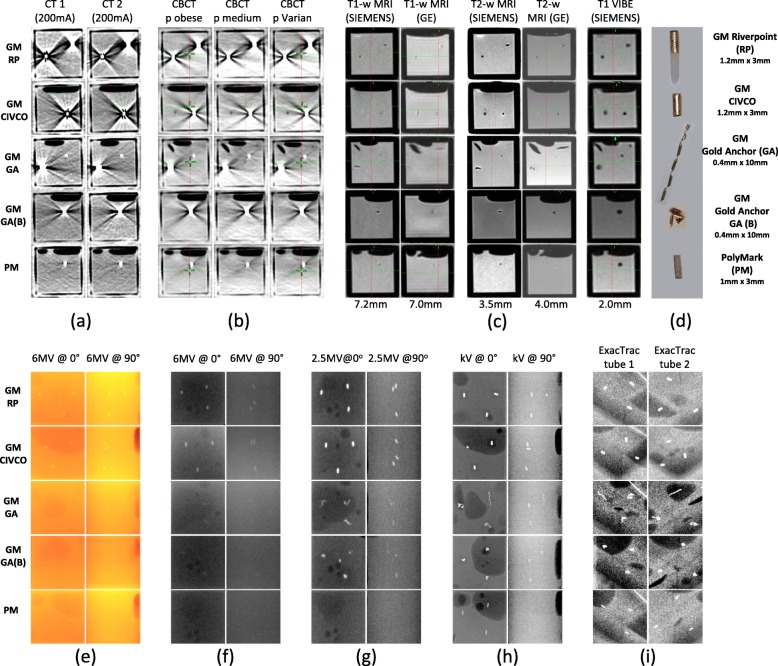
Cross-sectional/planar images of boxes with different FM acquired using different imaging modalities. **a**–**b** Volumetric X-ray scans, **c** MRI scans, and **e**–**i** Planar X-ray images. **d** Different fiducial markers used in this study, diameter x length

Figure 3, shows a summary of cross-sectional/planar views for all FM investigated (images cropped to show each box with FM) using different IGRT imaging modalities and different imaging protocols. Volumetric X-ray CT and CBCT and MRI scans are displayed in Fig. 3a--c, respectively. Figure 3e--i displays planar X-ray images, generated from integrated images of the TrueBeam 6 MV treatment field, 2.5 MV and 6 MV imaging beams or kV images acquired using the TrueBeam on-board imager or the Brainlab ExacTrac imaging systems.

As can be seen of Fig. 3a, b, all markers were clearly visible on CT and CBCT scans. CT scans were acquired using 120 kVp and 200 mA or 400 mA tube current. Changing tube current did not affect the visibility and artefact of different markers, therefore, only scans using 200 mA are presented in Fig. 3a. PM FMs were observed to have the least artefact on both CT and CBCT scans. It was also observed that gold marker edges were difficult to identify on CBCT, indicating more reconstruction and beam hardening artefact volume around the GM compared to CT scans. The MRI study presented in Fig. 3c shows that all FMs resulted in absence of signal and were observed as dark circles/ovals on T1w and T2w-MRI scans. In this study, a 3D Gradient Echo VIBE scans (recommended by the GA manufacturers) were also acquired. All FM were clearly visible on VIBE scans, however, the size of the markers were 4 --27 fold larger than their actual size, indicating the manifestation of MRI susceptibility artefacts, Table 1 and Fig. 3c.

In addition to the visual assessment of FM visibility, the magnitude of the FM image-contrast was also assessed using line profiles acquired through each FM in its surrounding gel environment on different volumetric imaging modalities. Additional file 1: Figure S3 displays profiles through 3 orthogonal planes (left-right (LR), anterior-posterior (AP) and superior-inferior (SI)) of CT and CBCT scans acquired of the FMs. The line profiles presented peaked at 3071 HU for gold markers with high Z values on CT scans (due to saturation of HU number scales) and at just below 7000 HU on CBCT scans. Small discrepancies were observed when comparing the left-right (LR), Anterior-Posterior (AP) and Superior-Inferior (SI) line profiles of the same marker due to the captured orientation of the marker on each tested plane and the reconstruction orientation. These discrepancies in HU affected both the peak values as well as the loss of signal (dip in HU) around the FMs i.e. artefacts due to shadowing. Additional file 1: Figure S3 also shows line profiles (dotted lines) obtained from patients’ CT and CBCT scans of prostate and FMs. No patient data was available for the GM RiverPoint. In-vivo line profiles of prostate and FM were in good agreement with phantom line profiles. No differences were found when comparing line profiles of different CT acquisitions (different scanners and different tube current). Similar results were observed when comparing line profiles from different markers within each box and from the consistency box (repeated marker box).

On EPID integrated planar images, Fig. 3e, only larger diameter GMs were clearly visible on both anterior-posterior (AP) and lateral projections. Similarly, on 6 MV planar images, Fig. 3f only GM RP and CIVCO were visible. All GM were visible on 2.5 MV planar images (Fig. 3g) while PM was not visible. As shown in Fig. 3h, all FMs were clearly visible on AP planar kV images but again, the PM FMs were not visible on lateral kV views and GM GA (line shape) had very poor visibility. All FMs were clearly visible on both ExacTrac high-resolution medio-lateral stereoscopic X-Ray images due to its inclined projection avoiding the PVC material, Fig. 3i. Specification of planar images and the optimized window level settings used for planar images qualitative analysis are presented in Additional file 1: Table (ST3).

### Quantitative assessment

#### CNR for 2D images

From our analysis of 2D images (Table 2), FMs that had CNR < 1 were not visible (**nv**) and 1 < CNR < 3 had poor visibility while 3 and higher were clearly visible. On EPID integrated images RP and CIVCO GMs had higher CNR compared to GAs. PM was not visible on both AP and LAT projections. Only RP and CIVCO GMs were visible on 6MV planar images, while with 2.5 MV beams GA FM (line and ball shape) were also visible but with lower CNR values. On kV images all markers were visible and the highest CNR observed (9.38) was for the AP view of PM, however PM also had the lowest CNR (2.35) on the lateral projection. All FMs were visible on ExacTrac Stereoscopic planar images with comparable CNR values for all markers.

**Table 2:** Contrast-noise ratio (CNR) for different fiducial markers

Planar image	RP	CIVCO	GA	GA (B)	PM
EPID integrated 6MV 200MU/min ET 3379 s					
@0^o^	8.36	15.48	*2.61*	*1.67*	**nv**
@90^o^	9.73	4.93	**nv**	3.18	**nv**
6MV 6MV MsE 3 MU					
@0^o^	6.04	8.33	**nv**	**nv**	**nv**
@90^o^	4.44	5.60	**nv**	**nv**	**nv**
2.5MV 2.5MV MsE 1.5 MU					
@0^o^	8.56	7.39	4.24	5.94	**nv**
@90^o^	4.15	4.51	*2.63*	3.59	**nv**
kV 0^o^: kVp 85,134 mA ET 112 s 90^o^: kVp 110,102 mA ET 147 s					
@0^o^	*2.64*	*2.09*	4.82	6.40	9.38
@90^o^	6.08	6.40	4.62	3.95	*2.35*
ExacTrac 212^o^: kVp 100,400 mA ET 100 s 149^o^: kVp 100,400 mA ET 100 s					
Tube1	*2.78*	3.42	*2.57*	*2.57*	3.17
Tube2	*2.60*	*2.47*	3.21	3.90	*2.97*

*MsE* MeterSet Exposure, ***nv*** not visible, *italic* poor visibility

#### CNR and artefacts on 3D scans

All FMs were visible in all volumetric scans. Volumetric CNR values are presented in Table 3. In this analysis, volumetric CNR values of < 1 were still visible and the values presented are for comparison purposes. CNR results of different FMs on CT scans with different slice thicknesses had a very similar range: [1.27 --1.61]. Compared to CT scans, the CNR values for CBCT were lower, range: [0.64 --1.27]. On MRI, CNR values were all above 1 for all the markers in all investigated acquisition sequences with a slight increase for PM (range: [1.88--2.56]) compared to other gold FMs. The lowest CNR values on MRI were observed for ball shape GA(B) range: [1.44--1.64]. For comparing results with 2D planar images, a 2D analysis of CNR is also presented in the Additional file 1: Gold fiducials, RP and CIVCO, had the highest 2D CNR values [5.47--2.43] while GA(B) and PM had the lowest [1.83 --2.79]. More variability was observed in 2D CNR values for the different FMs compared to the 3D values presented in Table 3.

**Table 3:** Contrast-noise ratio (CNR) for volumetric imaging and steak artefact index (SI) on X-ray volumetric imaging for different fiducial markers

Volumetric imaging	Visibility (CNR)						Artefact (SI)				
	RP	CIVCO	GA	GA (B)	PM		RP	CIVCO	GA	GA(B)	PM
CT (slice thickness (mm))											
0.625	1.46	1.49	1.53	1.54	1.47		67.78	110.9	61.04	67.51	25.57
1.25	1.61	1.46	1.44	1.22	1.52		86.92	52.81	65.20	59.64	66.24
2.5	1.27	1.23	1.42	1.34	1.52		53.35	47.19	36.88	46.15	11.63
CBCT (protocol)											
P Varian	1.17	0.96	0.99	0.92	0.79		33.16	22.03	33.81	13.86	19.55
P Medium	0.94	0.94	0.64	1.07	0.90		26.38	42.57	15.58	19.91	9.82
P Obese	1.00	0.84	0.85	1.16	1.27		66.08	58.28	47.48	75.81	36.88
MRI							FM size (mm^3^)				
						**P**	**13.57**	**13.57**	**5.03**	**5.03**	**9.42**
T1w	1.17	1.69	1.77	1.64	2.56		6.67	10.00	6.67	26.67	3.33
T2w (filtered)	1.95	1.87	1.54	1.45	1.88		13.33	20.00	16.67	23.33	13.33
VIBE	1.58	1.94	1.57	1.47	2.10		53.33	70.0	123.33	136.67	110.00

Note that CNR and SI values are calculated for each box as a contribution of the 3 FMs contained in each box while FM size in MRI corresponds to one FM to facilitate the comparison with their physical dimensions. **P** indicates the physical size of the markers calculated assuming the markers are perfect cylinders

As can be seen on Table 3, streak artefacts on CT and CBCT were similar for all gold markers, while the lowest SI values were found for PM. SI increased with reducing slice thickness for all FMs. Overall, SI values for GMs were lower in CBCT compared to CT scans.

### Patient data

**Fig. 4:**
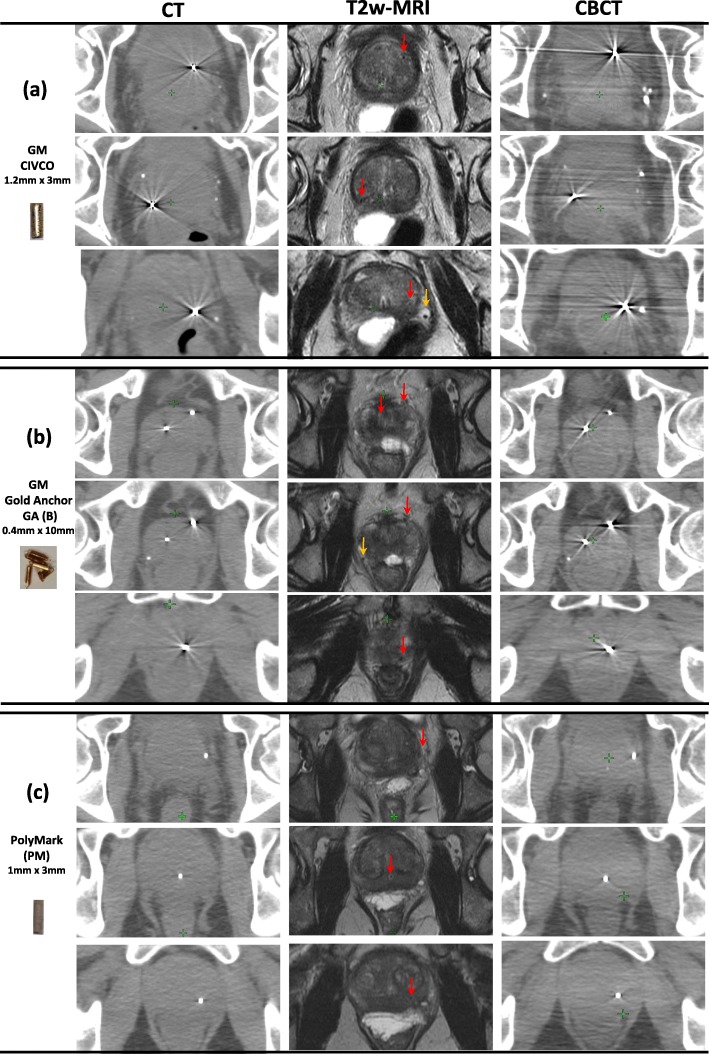
Prostate gland of three FM on three different patients as they appear on axial slices of CT/CBCT and T2w-MRI. Each patient had 3 fiducial markers. Patient 1 (**a**) GM CIVCO; routinely used in our institute for IGRT, patient 2 had GM GA (implanted as a ball shape) (**b**) and patient 3 had a PM polymer markers (**c**). FM indicated with red arrows on MRI and the orange arrows point to natural calcifications

Figure 4 shows axial views of the prostate gland of three patients with three different FMs on multimodal imaging typically employed for prostate cancer, i.e. CT, CBCT and T2w-MRI. The scanning parameters are presented in Additional file 1: Table ST2. Figure 4 demonstrates that the quality of CT and CBCT scans was very similar for the three patients, however there is noticeable variability in the appearance of the prostate on T2w MRI scans. All markers were clearly visible on CT and CBCT scans. Identifying CIVCO GMs on T2w MRI for patient 3 was challenging. CIVCO GMs also showed the most prominent artefact on CT and CBCT. The GM GA, observed on patient 2 scans, were implanted to form a ball shape. GA was visible on the three imaging modalities with fewer artefacts (CT SI = 90.90) compared to CIVCO GM (CT SI = 110.10). Polymer-based FM, PM, was the easiest to identify on MRI and had the least artefacts on CT (CT SI = 14.15) and CBCT.

In agreement with Fig. 4, Fig. 3a shows that all FMs implanted in the pelvic phantom had excellent visibility on CT and CBCT scans, regardless of their material or size. The most dominant artefacts on CT and CBCT scans were observed around the larger diameter GMs (CIVCO and RiverPoint). Smaller diameter GM GA also showed similar streak artefacts but these artefacts visually appeared to be less than those for larger diameter GMs. For patients shown in Fig. 4a, b, extra- and intra-prostatic calcifications (indicated by orange arrows on MRI) are also visible on scans. These naturally occurring calcifications are clearly visible on both CT and CBCT and some were also clearly visible on MRI, yet they did not introduce any artefacts.

The effect of different planning CT scan slice thickness on the visibility and artefacts is presented in Additional file 1: Figure S6. Overall, there was an increase in high HU values artefacts indicating an increase in streak artefact with reducing slice thickness.

Additional file 1: Figure S4 demonstrates that the gel used in this study is not prostate tissue equivalent for MRI scanning. As it is not possible to assess the contrast in signal intensities on raw line profile of the FMs and surrounding medium on patients’ MRI, both phantom and line profiles intensity values were normalized to values between 0 and 1. On Additional file 1: Figure S4, the left three panels show the normalized intensity values to facilitate the direct comparison of line profiles of the FMs/gel on the pelvic phantom and of FMs/prostate on patients’ MRI scans. It is clearly seen in this figure that line profiles from patients’ prostate are very noisy, showing that the prostate gland is highly inhomogeneous. The signal intensity of the marker is similar to that of the surrounding (inhomogeneous) prostate tissue highlighting the challenge experienced in identifying FMs on patients’ MRI scans. In this work, only T2w MRI line profiles are presented as it is the sequence most commonly used for prostate cancer patients. An example of line profiles on other MRI sequences of the Gold Anchor FM on different MRI sequences are also shown in Additional file 1: Figure S5. Similarly, Additional file 1: Figure S4 displays line profiles acquired from orthogonal planes of T2w-MRI scans.

## Discussion

In this study, a novel pelvic phantom was developed and utilized to assess the visibility and any associated artefacts of four different commercially available FMs on multi-modality imaging relevant to prostate cancer IGRT. Modifying the design of a previously reported phantom [24], this phantom was built to provide an adequate facsimile of a real patient pelvis for achieving the goals of this investigation. The phantom was designed with 3D printed boxes in the centre. These boxes were filled with gel to mimic the prostate gland and two parallel PVC rods were used to mimic bones in the pelvic region. The gel contained in the boxes had uniform volumes with only a few air bubbles at the surface of the gel. These air bubbles did not influence our study as none were presented near the fiducial markers in any of the boxes.

The visibility of different FMs and the artefacts they introduced in scans were assessed qualitatively through visual inspection and quantitatively by comparing contrast-to-noise ratios and steak artefact index. All FMs were clearly visible on volumetric imaging. Gold FM were brighter on CT and CBCT but they were surrounded by evident streak artefacts. Polymer fiducial markers were superior to gold markers for volumetric imaging as they introduced minimal artefacts on CT and CBCT and were clearly visible on CT, CBCT and on MRI. These observations were also supported by the quantitative analysis as CNR values were similar for PM and GMs, whereas PM boxes had lower SI index values compared to other markers. The superiority of PM on volumetric imaging is also confirmed by the retrospective patient data presented. On planar imaging, PM FMs were also clearly visible on planar kV and ExacTrac projections. However, PM FMs were not visible on EPID MV or on lateral kV planar images. Even when using the low-MV imaging beam (2.5 MV) option [29] that provided better image contrast for all gold FMs, PM visibility on planar images did not improve. When EPID verification or kV imaging was used for image guidance, GMs with large diameters were the most visible FMs on both LAT and AP planar images. Consequently, the choice between different fiducial markers in prostate cancer treated with external beam radiotherapy is highly dependent on the IGRT strategy adopted.

Riverpoint and CIVCO GM with larger diameter (d = 1.2 mm) created more streak artefact compared to GA (d = 0.4 mm). However, GA FM also showed artefacts that were similar to RiverPoint and CIVCO when used in ball shape (GA(B)). No differences in visibility or artefacts were observed when using a CT tube current of 200 mA compared to 400 mA or when scanning the phantom on different CT scanners. Using different CBCT acquisition protocols did not seem to affect the visibility on CBCT scans, however an increase in SI was observed when using the “obese” CBCT protocol (140kVp) compared to other CBCT protocols (125kVp). Employing VIBE scans on MRI, the FM appeared larger than actual physical size and compared to their size on other MRI sequences. This could be related to magnetic susceptibility artefact as a result of using gradient echo based imaging (VIBE) on MRI [30].

The line profiles across different FM provided an objective comparison of marker signals on CT, CBCT and MRI scans. However, some of the profiles did not capture the true peak HU values as the CT scanners were limited to a 12 bit output range and the peak HU exceeded the 3071 saturation value. As expected, higher peaked HU (elements with high atomic number) corresponded to more contrast in the image (more visibility) but also resulted in more image artefacts. Image reconstruction on CT scanners usually include a series of correction steps for beam hardening, scattered radiation and noise measurement. However, in the presence of metal implants these corrections may not be sufficient [31, 32]. The impact of metal artefacts/distortion of images varies depending on the type of radiation treatment and the size of the metallic implants, as well as the location of these implants relative to the treatment site [16]. Several metal artefact reduction (MAR) techniques have been developed in recent years to aid improving organ delineation and dose calculation in radiation therapy treatments [28–30]. Despite their effectiveness in removing/reducing artefacts, MAR techniques may also cause undesirable loss of detail around markers.

In their study, Chan et al. [23] compared the visibility and artefacts of different sizes of gold markers (solid and coiled) and PM markers on CT, CBCT, OBI-kV, ultrasound and MRI. The authors reported that GM with diameter < 0.75 mm were not visible in EPID MV images. In our study, this was also observed in EPID planar images with 6 MV beams but when using 2.5 MV beams both GA and GA(B) were visible (d = 0.4 mm). In line with our results, they concluded that GMs with larger diameters showed more artefacts and that the degree of artefact is also dependent on scanner and scanner settings. In their experimental setup, FMs were placed in a custom bolus phantom and Surgilube gel was used around the FMs to reduce air gaps. The Surgilube gel caused local artefact on T2w MRI, therefore only T1-w MRI scans were assessed [23]. In an earlier study using a cubical bolus phantom, Handsfield et al. compared gold, carbon and polymer FM using different imaging modalities [21]. They concluded that when kV imaging is used, polymer and carbon FM were preferred due to their reduced artefacts compared to GMs, but when MV imaging is used, gold markers may be necessary. Both studies [21, 23] utilized phantoms consisting of only one density (bolus material) which makes their results applicable for soft tissue only. In the present study, although not being prostate tissue equivalent on MRI (different mean and SD pixel intensities), the gel used did not interfere with the analysis conducted.

The pelvic phantom presented here provided an improvement to commonly used simplified phantoms for assessing prostate FMs. The CNR analysis of planar images revealed that CNR values were higher overall for AP planar images compared to LAT views (x-rays passing through high density PVC material mimicking bone). Therefore, assessment of FMs on simplified gel phantoms may not be sufficient for prostate cancer IGRT studies.

There has been several reports in the literature promoting the use of hydrogel based liquid fiducial (LFM) markers for CT and MRI due to their sufficient visibility and reduced artefacts compared to metallic markers, however there are also concerns regarding long term stability which is crucial in fractionated radiotherapy [33–35]. A recent study investigated a stable biodegradable liquid fiducial marker (BioXmark) with GA (99.5% Au + 0.5% Fe, d = 0.28 mm) and Visicoil (> 99.9% Au, d = 0.35 mm) GMs for MRI-guided imaging and proton therapy using a pancreas tissue-mimicking spherical gel phantom [25]. Schneider et al. reported that GA were better in terms of visibility but also induced more artefacts due to their iron content. For all solid FMs investigated, they observed a direct linear relationship between the potential visible size and artefact size. The authors discussed that, in contrast to GMs’ signal void that is caused by their effect on T2* of the surrounding tissue, liquid FMs cause signal void due to the absence of water protons. As a result, unlike GMs [22], no correlation between size and artefacts was noticed for liquid FMs and the markers with volumes (25--100 μL) had the highest visibility compared to 10 μL FM. Therefore, when a low level of image distortion is required, liquid markers are better than solid gold markers. The phantom employed also consisted of one density gel that was previously reported to be pancreatic tissue equivalent on 3 T MRI scans [36].

In this work, we have for the first time, reported on the use of a pelvis-mimicking phantom with bone equivalent material (PVC) and prostate tissue (gel) to study FMs’ visibility and artefacts on clinically relevant multi-modality imaging. There are a few limitations to this study. Firstly, the gel used was found to be tissue equivalent on X-ray imaging but not on MRI. Therefore, although it is a good approximation, the contrast (visibility) may not be equivalent to what is observed on MRI of prostate tissue. The homogeneous gel used in this study did not reflect the heterogeneity of prostate gland as captured on MRI and gels that are more representative of the prostate gland and prostate cancer will need to be developed [27]. Moreover, the limited lifetime of about 3–4 weeks (when refrigerated) of the gel used in this study does not allow for a long-lasting phantom. Therefore, further research into finding non-biological tissue equivalent materials is required to provide durable phantoms that are easy to store and that can maintain consistent characteristics. The methodology reported here can also be extended to other types of FMs [21, 32], different imaging modalities and to study naturally occurring calcification for image guidance for prostate cancer patients which are very common in and around the prostate gland. A recent observational study of 254 prostate cancer patients showed that 85% of these patients had calcifications that were detectable on pre-treatment planning CT scans [37]. 99% the calcifications that were detected on CT were also detected on CBCT and remained visible at the end of radiotherapy course [37].

## Conclusion

In this work we have reported on a detailed study of five different commercially available FMs using eight different imaging modalities commonly used as part of the radiotherapy process. By developing a novel phantom to test the FMs, quantitative analysis of the visibility and the impact of artefacts on the image have been determined. This data has clearly shown that the choice of FM employed is dependent on the overall imaging strategy used. If volumetric imaging using CT, CBCT and MRI is used, then the polymer marker was shown to have the least significant artefacts while maintaining good visibility on the images. If MV verification imaging is used, then the gold FMs are required, with the larger diameter 1.3 mm being the most visible. However, these result in more significant imaging artefacts in the planning CT images. The phantom that has been developed was shown to be a versatile tool for the characterization of the different FMs in different imaging modalities. The use of gelatine provided a good representation of prostate on CT scans, but further development is required to accurately mimic MRI variations.

## Supplementary information

**Additional file 1 **: **Figure S1.** Typical IGRT workflow used for EBRT with CBCT. **Figure S2.** Distribution of CIVCO FMs in 6 consecutive patients in 3D, each colour represents a different FM location and the solid circle represents the average position for each marker. **Table ST1.** FM position consistency over a four weeks period. **Table ST2.** Scanners and scanning parameters and specifications patients’ volumetric imaging for patients presented in Fig. 4 of the main manuscript. **Table ST3*.*** Image specification and window levels settings used for planar images presented on Fig. 3. **Figure S3.** Intensity profiles showing each fiducial and surrounding intensities on CT (left) and CBCT scans using the pelvic phantom (red solid lines) and from prostate cancer patient with matching FMs (black dotted). **Figure S4.** Intensity profiles showing each fiducial and surrounding intensities on MRI scans using the pelvic phantom (red solid lines) and prostate cancer patients (black dotted lines). Original MRI signal intensity values on the left and normalized values on the right. **Figure S5.** Line profiles across corresponding axial slices of one of the boxes with Gold Anchor FMs and surrounding intensities on different sequences of MRI scans. **Figure S6.** Axial CT slices of boxes with different FM acquired using different CT slice thickness and the corresponding contrast-to-noise ratio (CNR) and HighHU artefact and LowHU artefacts. The CNR value for each FM were calculated using the Eclipse TPS. Background HU for gel (Mean±SD) were; 52.8 ± 33.6 for CT with 0.625 mm slice thickness, 52.8 ± 29.3 for CT with 1.25 mm scans and 50.5 ± 20.8 for CT with 2.5 mm slice thickness. The HighHU and LowHU artefacts were calculated relative to background (Gel) HU ± 3SD. * Indicates the presence of artefact from the box wall leading to unreliable measurements.

## Data Availability

The datasets used and/or analysed during the current study are available from the corresponding author on reasonable request.

## Abbreviations

CBCT: Cone beam CT
CNR: Contrast-noise-ratio
DIL: dominant intra-prostatic legion
EBRT: External beam radiotherapy
EPID: Electronic portal imaging device
FM: Fiducial marker
GA: Gold Anchor
GM: Gold fiducial marker
HU: Hounsfield unit
IGRT: Image guided radiotherapy
IMRT: Intensity modulated radiotherapy
MAR: Metal artefact reduction
PM: Polymer based fiducial marker
ROI: Region of interest
SABR: Stereotactic ablative body radiation therapy
SI: Streak artefact index
SPORT: Stereotactic prostate RT in high-risk localised prostate cancer with or without elective nodal irradiation
VMAT: Volumetric modulated arc therapy
