# Supplementary material for: Fiducial markers visibility and artefacts in prostate cancer radiotherapy multi-modality imaging
Source: Radiat Oncol. 2019 Dec 26;14:237. doi: 10.1186/s13014-019-1447-1 (PMC6933910; doi:10.1186/s13014-019-1447-1)
Supplement: Supplementary file 1 — Additional file 1 : Figure S1. Typical IGRT workflow used for EBRT with CBCT. Figure S2. Distribution of CIVCO FMs in 6 consecutive patients in 3D, each colour represents a different FM location and the solid circle represents the average position for each marker. Table ST1. FM position consistency over a four weeks period. Table ST2. Scanners and scanning parameters and specifications patients’ volumetric imaging for patients presented in Fig. 4 of the main manuscript. Table ST3. Image specification and window levels settings used for planar images presented on Fig. 3. Figure S3. Intensity profiles showing each fiducial and surrounding intensities on CT (left) and CBCT scans using the pelvic phantom (red solid lines) and from prostate cancer patient with matching FMs (black dotted). Figure S4. Intensity profiles showing each fiducial and surrounding intensities on MRI scans using the pelvic phantom (red solid lines) and prostate cancer patients (black dotted lines). Original MRI signal intensity values on the left and normalized values on the right. Figure S5. Line profiles across corresponding axial slices of one of the boxes with Gold Anchor FMs and surrounding intensities on different sequences of MRI scans. Figure S6. Axial CT slices of boxes with different FM acquired using different CT slice thickness and the corresponding contrast-to-noise ratio (CNR) and HighHU artefact and LowHU artefacts. The CNR value for each FM were calculated using the Eclipse TPS. Background HU for gel (Mean±SD) were; 52.8 ± 33.6 for CT with 0.625 mm slice thickness, 52.8 ± 29.3 for CT with 1.25 mm scans and 50.5 ± 20.8 for CT with 2.5 mm slice thickness. The HighHU and LowHU artefacts were calculated relative to background (Gel) HU ± 3SD. * Indicates the presence of artefact from the box wall leading to unreliable measurements. [file 13014_2019_1447_MOESM1_ESM.pdf]

Supplementary Material

Fiducial Markers Visibility and Artefacts in Prostate Cancer Radiotherapy Multi-modality Imaging

SO Osman, E Russell, RB King, K Crowther, S Jain, C McGrath, AR Hounsell, KM Prise and CK McGarry

Figure S1

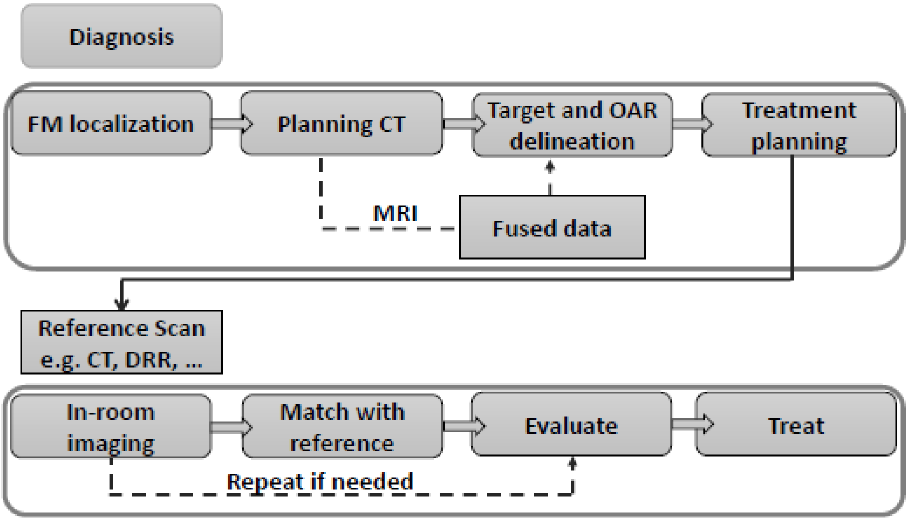

Figure S1: Typical IGRT workflow used for EBRT with CBCT.

Figure S2

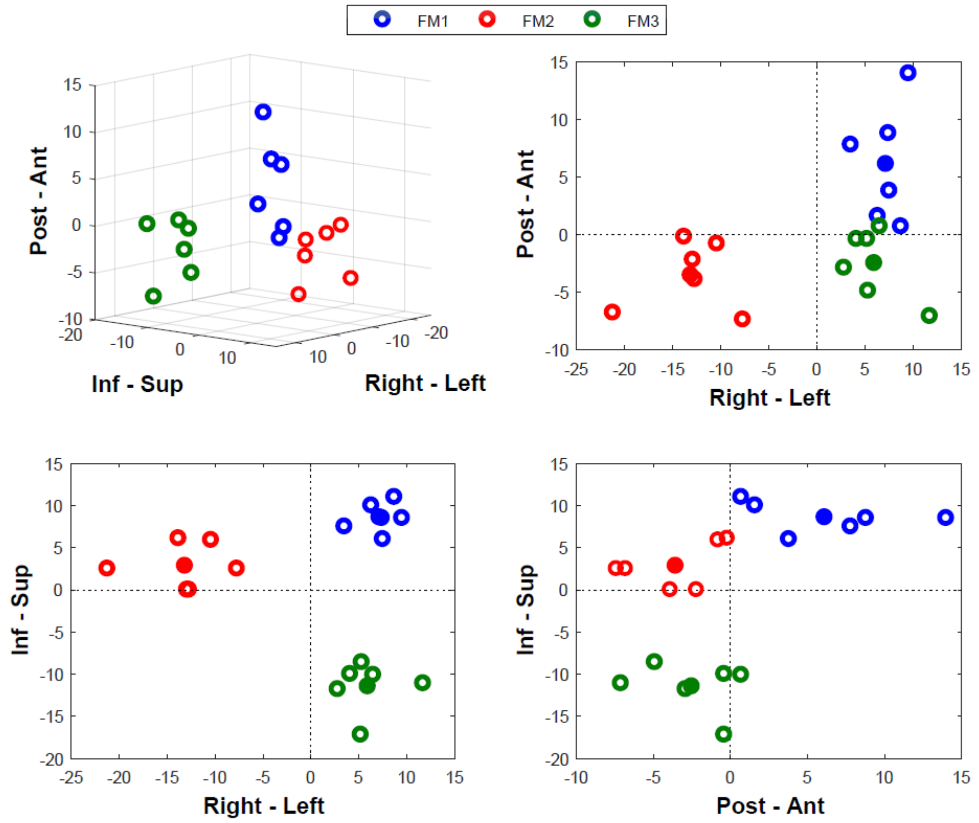

***Figure S2**: Distribution of CIVCO FMs in 6 consecutive patients in 3D, each colour represents a different FM location and the solid circle represents the average position for each marker.*

The boxes were stored in a fridge (2-4◦C). Results from the retrospective analysis of patient CT images are shown in supplementary figure S2. The marker distribution within each patient was observed to be consistent allowing the markers to be considered as three groups. Each colour in the plots represents one of the three groups of FMs. Open circles indicate the position of an FM within a patient, relative to the average position of the three FMs for that patient, while the solid circles indicate the average overall x, y and z position of each of the three FMs. These average positions were subsequently utilised during the preparation of the gel-filled cubes used in the phantom investigation.

Table ST1 FM position consistency over a four weeks period.

Date	**Difference from baseline** (mm)		
**Week 0 (Baseline)**	x	y	z
Left/Ant/Sup Corner			
Marker 1	0.0	0.0	0.0
Marker 2	0.0	0.0	0.0
Marker 3	0.0	0.0	0.0
**Week 1**			
Left/Ant/Sup Corner			
Marker 1	-0.2	0. 3	0.0
Marker 2	-0.2	0. 2	0.0
Marker 3	-0.2	0.2	-0.1
**Week 2**			
Left/Ant/Sup Corner			
Marker 1	-0. 3	0.3	0.1
Marker 2	-0.2	0.2	0.1
Marker 3	-0.3	0.2	0.1
**Week 3**			
Left/Ant/Sup Corner			
Marker 1	-0.1	0.9	0.4
Marker 2	-0.1	0.6	0.1
Marker 3	0.0	0.6	0.0
**Week 4**			
Left/Ant/Sup Corner			
Marker 1	0.0	0.1	0.8
Marker 2	-0.1	0.6	0.6
Marker 3	0.0	0.7	0.2

CT	Manufacturer	Model	Scan Type	Slice thickness (mm)	Pixel spacing (mm)	Focal spot (mm)	kVp	Tube current		FOV (mm)		
1	GE	Optima CT580	HELICAL	2.5	0.98	1.2	120	mA modulation		500		
2	GE	Optima CT580	HELICAL	2.5	0.98	1.2	120	mA modulation		500		
3	GE	Optima CT580	HELICAL	2.5	0.98	1.2	120	mA modulation		500		
**CBCT**	**Manufacturer**	**Model**	**Scan Type (Pelvis)**	**Slice thickness (mm)**	**Pixel spacing (mm)**	**Focal spot (mm)**	**kVp**	**Tube current (mA)**	**Exposure (mAs)**	**FOV (mm)**	**kV Filter**	**Bowtie**
1	Varian	TrueBeam	Varian	2	0.91	1	125	80	1049	464.9	Titanium	Half fan
2	Varian	TrueBeam	Varian	2	0.91	1	125	80	1072	464.9	Titanium	Half fan
3	Varian	TrueBeam	Varian	2	0.91	1	125	80	1074	464.9	Titanium	Half fan
**MRI**	**Manufacturer**	**Model**	**Sequence**	**Slice thickness (mm)**	**Slice gap (mm)**	**TR/TE (ms)**	**ETL**	**Flip angle (°)**	**Acquisition matrix**	**FoV (mm)**	**Receiver bandwidth (Hz/px)**	**Number of averages**
1	GE	SIGNA Explorer 1.5T	2D T2 TSE	3	0	7083/118	26	90/160	384×224	250	108.5	1.5
2	GE	SIGNA Explorer 1.5T	2D T2 TSE	3	0	7083/117	26	90/160	384×224	250	108.5	2
3	GE	SIGNA Explorer 1.5T	2D T2 TSE	3	0	7083/117	26	90/160	384×224	250	108.5	2

Table ST2: *Scanners and scanning parameters and specifications patients' volumetric imaging for patients presented in Figure 4 of the main manuscript.*

Planar imaging modality	Window level		Mode	bits allocated	viewing platform
	lower level	upper level			
**6MV@0°**	0	0.03	Dosimetry continuous	16	Eclipse TPS
**6MV@90°**	0	0.02	Dosimetry continuous	16	Eclipse TPS
**6MV@0°**	63181	65210	HighRes single	16	Eclipse TPS
**6MV@90°**	63834	65299	HighRes single	16	Eclipse TPS
**2.5MV@0°**	63183	65371	HighRes single	16	Eclipse TPS
**2.5MV@90°**	63355	65503	HighRes single	16	Eclipse TPS
**kV@0°**	53073	60274	DynamicGain single	16	Eclipse TPS
**kV@90°**	249837	251148	DynamicGain single	16	Eclipse TPS
**ExacTrac**	35	255	Xray	8	MATLAB
**ExactTrac**	75	255	Xray	8	MATLAB

Table ST3: *Image specification and window levels settings used for planar images presented on Figure (3).*

Figure S3

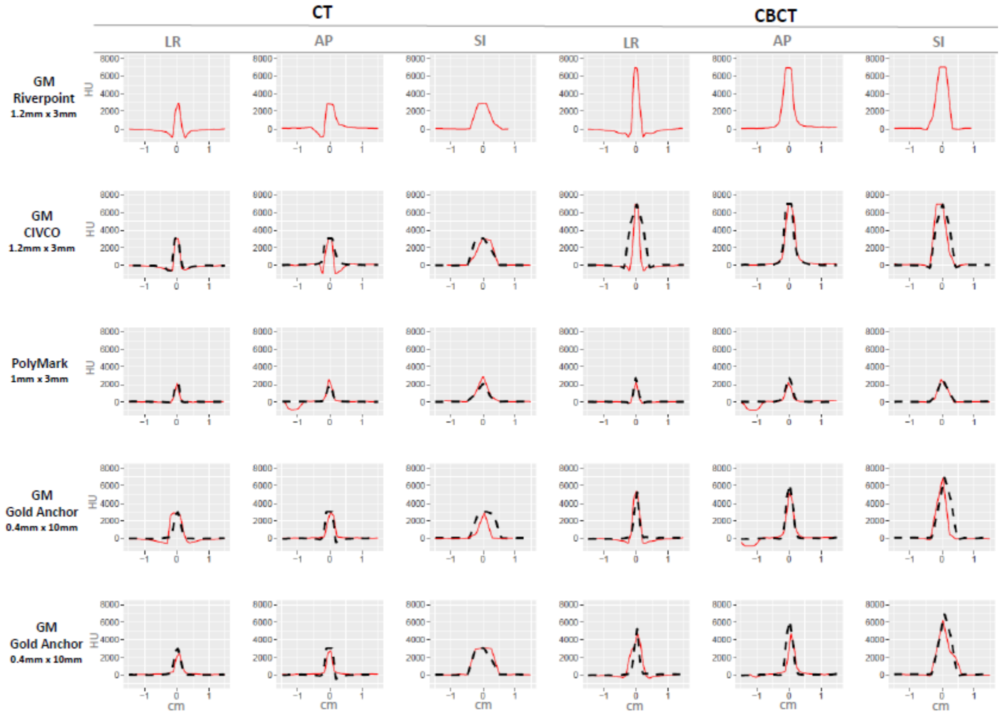

***Figure S3**:* Intensity profiles showing each fiducial and surrounding intensities on CT (left) and CBCT scans using the pelvic phantom (red solid lines) and from prostate cancer patient with matching FMs (black dotted).

Figure S4

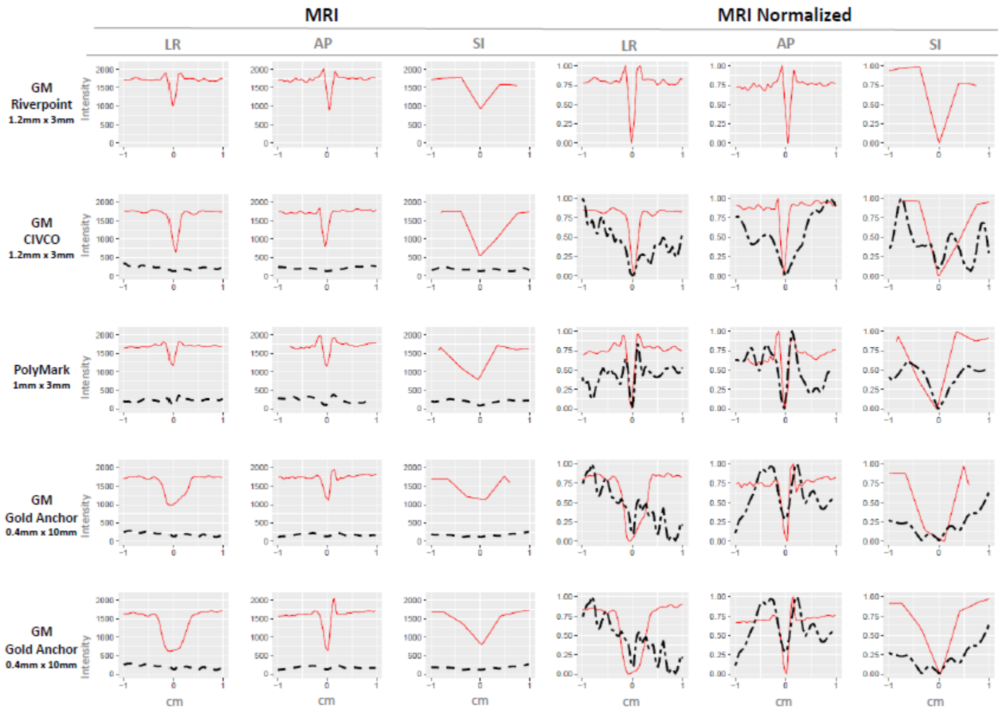

***Figure S4**:* Intensity profiles showing each fiducial and surrounding intensities on MRI scans using the pelvic phantom (red solid lines) and prostate cancer patients (black dotted lines). Original MRI signal intensity values on the left and normalized values on the right.

Figure S5

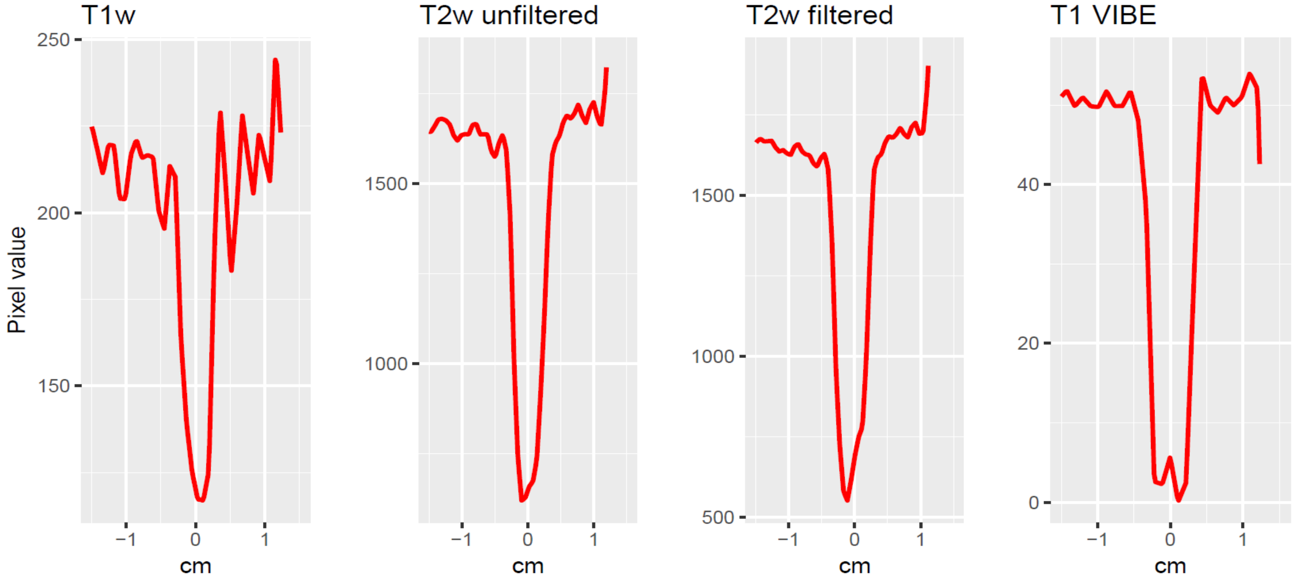

***Figure S5**: line profiles across corresponding axial slices of one of the boxes with Gold Anchor FMs and surrounding intensities on different sequences of MRI scans.*

Figure S6

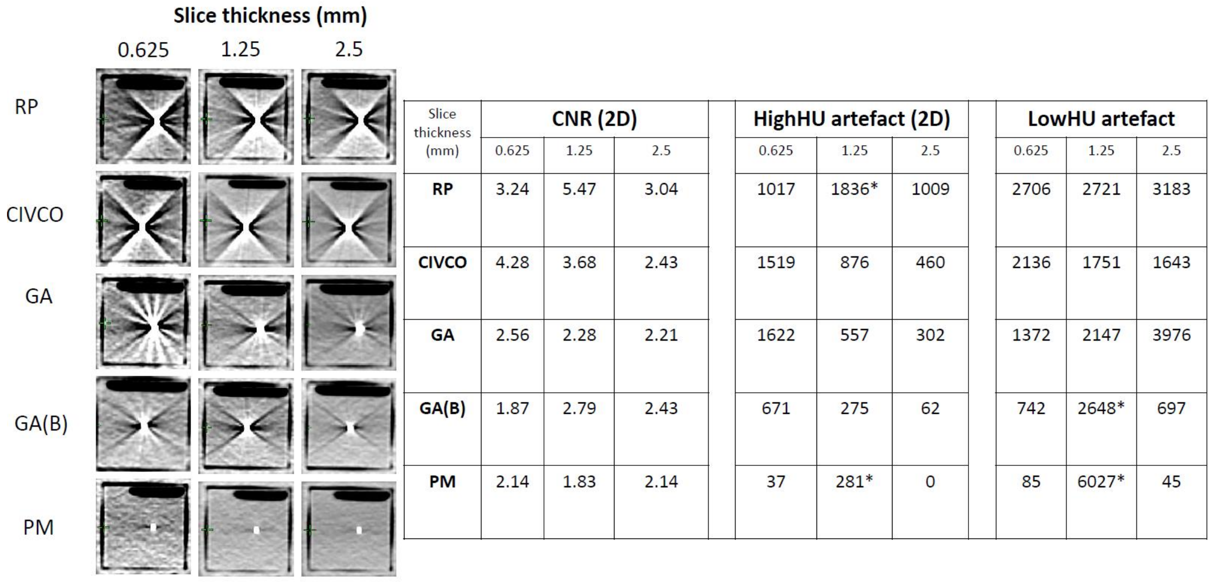

Slice thickness (mm)	CNR (2D)				HighHU artefact (2D)				LowHU artefact		
	0.625	1.25	2.5		0.625	1.25	2.5		0.625	1.25	2.5
**RP**	3.24	5.47	3.04		1017	1836*	1009		2706	2721	3183
**CIVCO**	4.28	3.68	2.43		1519	876	460		2136	1751	1643
**GA**	2.56	2.28	2.21		1622	557	302		1372	2147	3976
**GA(B)**	1.87	2.79	2.43		671	275	62		742	2648*	697
**PM**	2.14	1.83	2.14		37	281*	0		85	6027*	45

Figure S6: axial CT slices of boxes with different FM acquired using different CT slice thickness and the corresponding contrast-to-noise ratio (CNR) and HighHU artefact and LowHU artefacts. The CNR value for each FM were calculated using the Eclipse TPS. Background HU for gel (Mean±SD) were; 52.8 ± 33.6 for CT with 0.625 mm slice thickness, 52.8 ± 29.3 for CT with 1.25 mm scans and 50.5 ± 20.8 for CT with 2.5 mm slice thickness. The HighHU and LowHU artefacts were calculated relative to background (Gel) HU ± 3SD. * Indicates the presence of artefact from the box wall leading to unreliable measurements.
